# Effective autodissemination of pyriproxyfen to breeding sites by the exophilic malaria vector Anopheles arabiensis in semi-field settings in Tanzania

**DOI:** 10.1186/1475-2875-13-161

**Published:** 2014-04-29

**Authors:** Dickson Lwetoijera, Caroline Harris, Samson Kiware, Stefan Dongus, Gregor J Devine, Philip J McCall, Silas Majambere

**Affiliations:** 1 Environmental Health and Ecological Sciences Thematic Group, Ifakara Health Institute, P.O. Box 53, Ifakara, Tanzania; 2 Vector Biology Department, Liverpool School of Tropical Medicine, Pembroke Place, Liverpool L3 5QA, UK; 3 Division of Biomedical and Life Sciences Lancaster University, Bailrigg, Lancaster LA1 4YW, UK; 4 Department of Mathematics, Statistics and Computer Science, Marquette University, Milwaukee WI-53201-1881, USA; 5 Department of Epidemiology and Public Health, Swiss Tropical and Public Health Institute, Socinstr.57, P.O. Box, CH-4002 Basel, Switzerland; 6 QIMR Berghofer Medical Research Institute, Brisbane, Queensland, Australia

**Keywords:** Autodissemination, Pyriproxyfen, *Anopheles arabiensis*, Malaria, Africa, Vector control, Semi-field system, Clay pots

## Abstract

**Background:**

Malaria vector control strategies that target adult female mosquitoes are challenged by the emergence of insecticide resistance and behavioural resilience. Conventional larviciding is restricted by high operational costs and inadequate knowledge of mosquito-breeding habitats in rural settings that might be overcome by the juvenile hormone analogue, Pyriproxyfen (PPF). This study assessed the potential for *Anopheles arabiensis* to pick up and transfer lethal doses of PPF from contamination sites to their breeding habitats (*i.e.* autodissemination of PPF).

**Methods:**

A semi-field system (SFS) with four identical separate chambers was used to evaluate PPF-treated clay pots for delivering PPF to resting adult female mosquitoes for subsequent autodissemination to artificial breeding habitats within the chambers. In each chamber, a tethered cow provided blood meals to laboratory-reared, unfed female *An. arabiensis* released in the SFS. In PPF-treated chambers, clay pot linings were dusted with 0.2 – 0.3 g AI PPF per pot. Pupae were removed from the artificial habitats daily, and emergence rates calculated. Impact of PPF on emergence was determined by comparing treatment with an appropriate control group.

**Results:**

Mean (95% CI) adult emergence rates were (0.21 ± 0.299) and (0.95 ± 0.39) from PPF-treated and controls respectively (*p* < 0.0001). Laboratory bioassay of water samples from artificial habitats in these experiments resulted in significantly lower emergence rates in treated chambers (0.16 ± 0.23) compared to controls 0.97 ± 0.05) (*p* < 0.0001). In experiments where no mosquitoes introduced, there were no significant differences between control and treatment, indicating that transfer of PPF to breeding sites only occurred when mosquitoes were present; *i.e.* that autodissemination had occurred. Treatment of a single clay pot reduced adult emergence in six habitats to (0.34 ± 0.13) compared to (0.98 ± 0.02) in the controls (*p <* 0.0001), showing a high level of habitats coverage amplification of the autodissemination event.

**Conclusion:**

The study provides proof of principle for the autodissemination of PPF to breeding habitats by malaria vectors. These findings highlight the potential for this technique for outdoor control of malaria vectors and call for the testing of this technique in field trials.

## Background

Malaria remains one of mankind’s leading public health challenges and a major economic burden for the developing nations where it is endemic. Disproportionately, 80% of all malaria cases and 90% deaths occur in Africa [1]. The World Health Organization (WHO) continues to recommend a range of combined strategies for malaria prevention with vector control, primarily through the use of insecticide-treated bed nets (LLINs) and indoor residual insecticide spraying (IRS), a key component of those strategies [2-4]. Despite great progress in reducing malaria transmission in Africa over the past decade, the future use of both of these interventions, and indeed any approach that relies on chemical insecticides, is seriously threatened by the emergence and ongoing spread of insecticide resistance [5-8]. Moreover, LLINs and IRS target only vectors that are active indoors, and even in areas where this has been successful, malaria transmission by outdoor biting and outdoor resting vector populations of *Anopheles arabiensis* and *Anopheles funestus* remains a serious public health challenge [9,10]. Effective sustainable tools or approaches with proven impact on outdoor biting and resting vector populations have yet to be developed.

Targeting the aquatic larval stages of the vector with conventional insecticides (larviciding), as a complement to LLINs and IRS, can be an effective method to suppress vector density [11], but it is limited by the difficult task, and high cost, of identifying and treating sufficient mosquito breeding habitats to impact the vector population [12,13]. WHO recommendations limit the use of larviciding to settings where larval habitats are few, findable, and easy to map and treat; typically this restricts larviciding to urban settings [14]. In rural settings where breeding habitats are abundant in number and character, this is a far greater challenge for which novel approaches are urgently needed.

Pyriproxyfen (PPF) is a juvenile hormone analogue (JHA) that interrupts normal development and metamorphosis of targeted mosquitoes [15]. Highly potent in terms of activity and specificity, it has low toxicity and a high margin of safety to non-target organisms [16] and to date, there has been no evidence of PPF resistance in any mosquito [17]. For effective mosquito control, WHO recommends a PPF dosage limit of 50 ppb, an extremely low level considering the maximum permissible level in drinking water is 300 ppb [18]. PPF can be delivered in formulations that persist in treated aquatic habitats for up to six months under field conditions [19,20]. PPF also has an additional unique benefit, termed autodissemination, which is defined as the ability of adult mosquitoes to pick up PPF from treated solid surfaces, retain and transfer it to breeding habitats in sufficient quantities to contaminate those habitats, rendering them unproductive either by killing larvae or preventing pupae from emerging to adults [21].

The few studies demonstrating the potential of autodissemination of PPF in vector control have been limited to the *Aedes* vectors of dengue and chikungunya viruses [21,22]. Small field trials in urban settings in Peru and Italy, against *Aedes aegypti*[21] and *Aedes albopictus*[22] respectively, resulted in significant adult emergence inhibition in treated areas. Many aspects of the biology of these *Aedes* species, such as their aggressive feeding, skip-oviposition (distributing portions of each egg batch in multiple habitats) and preference for relatively small volume man-made containers as breeding habitats, undoubtedly contribute to the prospect for exploiting autodissemination in urban control programs for dengue and chikungunya [19,21,22] and fabrication of efficient PPF contamination sites/stations [22,23]. The outdoor-active *Anopheles spp.* that transmit malaria in rural Africa breed in a wide variety of breeding habitats, ranging in size and character and across much larger areas [24] and are a much greater challenge for this approach.

This study reports on the first experiments undertaken in a large semi-field system in Tanzania, evaluating the potential of PPF autodissemination for control of *An. arabiensis* and probably other African malaria vectors. Here, the results of controlled experiments quantifying the efficacy of clay pots, a simple inexpensive PPF contamination station, for delivering PPF to resting adult female *Anopheles arabiensis* at levels that prevent emergence at untreated breeding habitats are presented, demonstrating for the first time that, in principle autodissemination of PPF can occur at operationally effective rates in an *Anopheles arabiensis*, an efficient African malaria vector.

## Methods

### Study site

This study was carried out at Kining’ina village (8.11417 S, 36.67484 E), in rural southern Tanzania, between May 2012 and October 2013 inside a semi-field system (SFS). Details of the design and use of this SFS have been provided previously [25,26]. Briefly, the SFS is an outdoor construction with mesh walls 4.53 m high, measuring 552.96 m^2^ in total area but partitioned into six separate chambers each measuring 9.6 × 9.6 m. The concrete floors of the chambers were filled to a depth of 40 cm with local soil, and vegetation was allowed to grow naturally from the seeds therein. Although the SFS had six chambers, only four chambers were used for the experiments. A simple mud hut (1.75 m × 1.5 m, 2 m high) was built within each chamber to provide a shelter for a tethered cow bait, and possible resting location for mosquitoes. The simple mud hut was built to mimic the shelters used by communities to keep cows and not to represent an indoor set up.

### Mosquitoes

All sets of experiments were performed using insectary-reared unfed mated *An. arabiensis* females aged 3 – 9 days post eclosion. It was assumed that mosquitoes at this age would have mated [27]. The *An. arabiensis* colony was established in March 2010, originating from individuals collected in Lupiro village within the Kilombero valley. It is reared routinely inside a semi-field system (SFS) under natural temperature and 12: 12 h light: dark photoperiod of that area. Larvae were fed Tetramin^®^ fish food and adults maintained on human blood and 6% glucose solution. Mosquitoes were starved of sugar and water six hours prior to release in the experiments.

### Experimental procedures and study design

Five experiments were conducted between May 2012 and September 2013: first, to investigate the existence of PPF autodissemination from PPF-treated clay pots to the breeding habitats by contaminated mosquitoes; second, to confirm that the observed PPF contamination at the experimental breeding habitats was mosquito-borne; third, to investigate mosquito resting site preferences inside the SFS; fourth, to measure the proportion of mosquitoes resting inside the clay pots that were subsequently able to contaminate oviposition sites; fifth, to measure amplification of autodissemination from limited numbers of treatment points to a greater number of breeding sites.

### Experiment 1: Evaluation of PPF-treated clay pots for the delivery of pyriproxyfen to resting adult female mosquitoes for subsequent autodissemination

In every replicate of this experiment, 1500 – 5000 adult female *An. arabiensis* were released inside an SFS chamber, where a cow was provided for blood feeding, clay pots as resting sites during egg development, and water containers as oviposition sites. Clay pots have been used for sampling wild *An. arabiensis*, as adult females of this and other species will rest within these and similar vessels [28,29].

**Figure 1:**
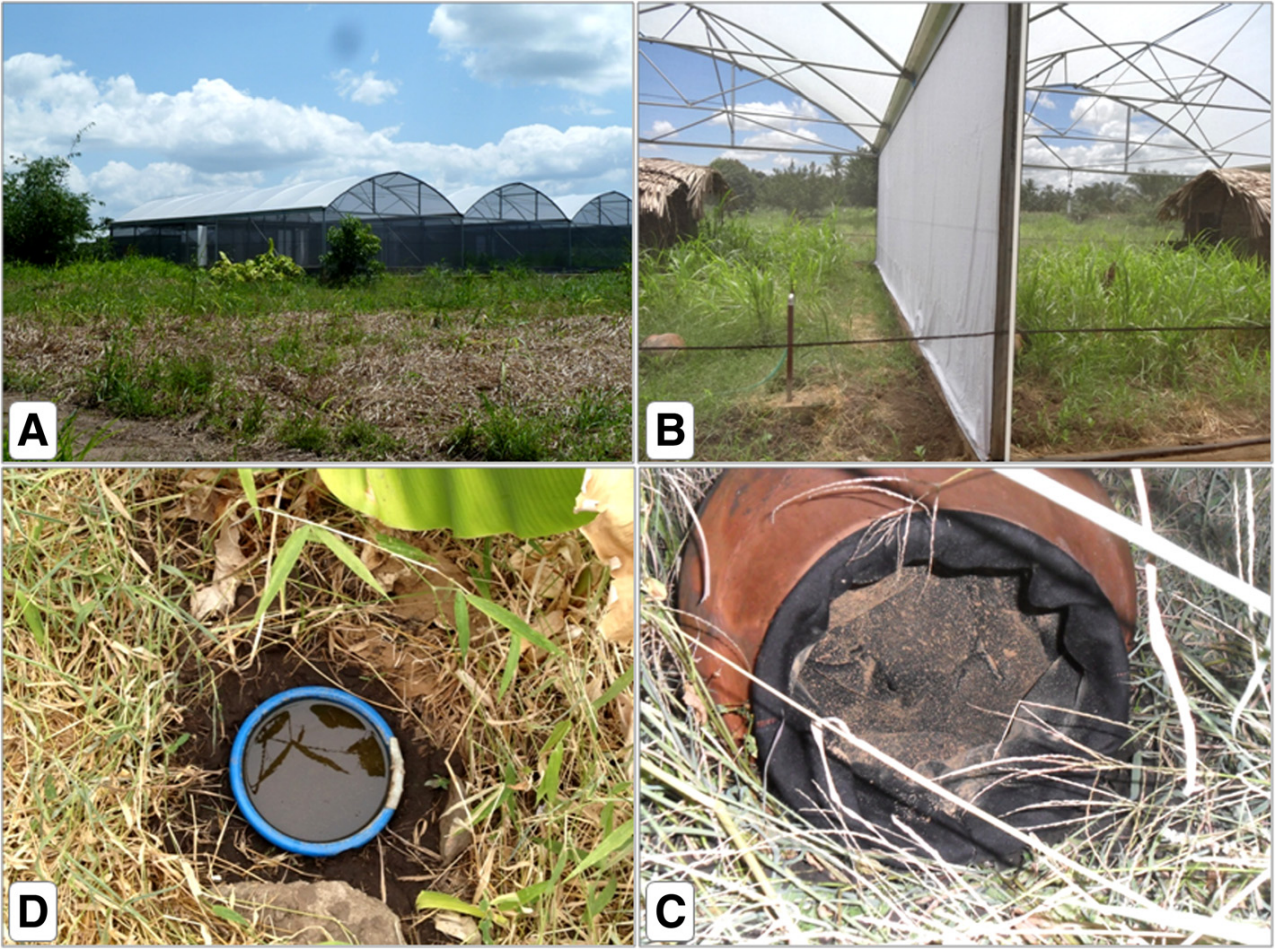
(A) The semi field system used in experiments; (B) adjoining chambers with huts for housing bait cows visible in each; (C), Pyriproxyfen (PPF)-treated cloth interior of a clay resting pot placed on the ground within a chamber; (D) plastic basin sunk in the ground within a chamber to provide the artificial habitat for egg laying.

Eight 10 L clay pots were placed on the ground: 5 around the perimeter of the SFS chamber and the other 3 around the walls of the mud hut. Each pot was lined with black cotton that had been dampened with water and dusted with PPF powder (0.2 – 0.3 g AI per clay pot; Sumilarv^®^, Sumitomo Chemical Co. Ltd., Japan). Dusting was done by unevenly sprinkling 2 – 3 g of 10% AI PPF powder over all surface of dampen cotton cloth using a makeup/painting blush. The cotton cloth was treated with PPF after being attached inside around the circumference of clay pot using 3 mm aluminium wire to ensure maximum containment of PPF powder (Figure 1C). Pots were allowed to dry for 24 hours, facilitating the PPF powder to attach lightly to the fabric while not hindering its pickup by mosquitoes that contacted it. Two identical artificial breeding habitats (2.5 L plastic basins, 21 cm in diameter; filled with 250 g of soil and 2 L of water; water levels were replenished as required) were buried with the rim at ground level, 5 m apart and between 1 and 8 m from clay pots (Figure 1). At the start of each experiment, 1,500 – 5,000 unfed female mosquitoes (aged between three and nine days post eclosion and caged with males until used) were released at 18.00 hrs. A cow, tethered inside the mud hut, was available for the first three days to permit blood feeding.

The experiment was allowed to run for 25 days following release of the mosquitoes, to allow 10 days until the first pupae developed and a further 15 days to harvest all pupae from the artificial aquatic habitats that successively developed from eggs laid by released mosquitoes. The breeding habitats were visually examined daily for the presence of eggs and larvae to confirm if mosquitoes visited the habitats. Each day, pupae developed from deposited eggs were removed, counted and transferred to an insectary where they were maintained under the cage in cups containing water from the habitats until they emerged as adults or died.

Control experiments were run simultaneously in a separate chamber using an identical protocol but without any PPF application to the cotton lining of the clay pots. A total of six replicates of both treatment and control experiments were run, over a period of 6 months. Treatment and control chambers were separated by a distance of 3.2 m and, to avoid PPF contamination of the control chamber, the same SFS chambers were used in all replicates for treatment and control. Of importance, control and treatment were not rotated but fixed between chambers, when one replicate was on-going in a pair of control and treatment chambers; the other pair of control and treatment chambers was put into uses. Where control and treatment chambers were adjacent to each other, a panel of white cloth was mounted on one side of partition net to prevent movement of PPF particles between chambers. A break of at least seven days between replicates minimized the chance of any mosquitoes surviving from the previous replicate. PPF contamination between replicates was minimized by spraying the chamber structure, the hut and vegetation with water, new plastic basins were used and cow were thoroughly cleaned by washing with only water without soap before each replicate. Successful contamination and dissemination was evaluated by comparing the differences in pupal mortality and emergence inhibition from the basins between treated experiments and controls.

PPF contamination of water in the experimental breeding habitats was investigated further by two methods. First, immediately after recording first stage larvae in the breeding habitats (typically 5–8 days after mosquito release), three 150 ml water sub-samples were collected from each habitat and transferred to separate 250 ml glass beakers. Twenty 2^nd^ or 3^rd^ instar *An. arabiensis* larvae taken from the laboratory colony (*i.e.* fresh uncontaminated individuals) were placed in each beaker and daily mortality and emergence rates were recorded until all were dead or had emerged as adults. The procedures were repeated twice, i.e. only in two consecutive experiments of the six experimental replicates.

In a second bioassay, at the termination of each experimental replicates (*i.e.* day 25 following initial introduction of adult females) and after pupation of all larvae and removal of all pupae, 250 second or third instar *An. arabiensis* larvae taken from the laboratory colony (*i.e.* fresh uncontaminated individuals) were introduced in each breeding habitat (assumed to be contaminated with PPF from previously released adults) and daily mortality and emergence was recorded until all were dead or had emerged as adults. The procedures were repeated twice, i.e. only in two consecutive experiments of the six experimental replicates.

### Experiment 2: Confirmation that pyriproxyfen contamination of breeding habitats was mosquito-borne

To examine whether the PPF impact on adult emergence from the breeding habitats observed in the previous experiment might have resulted from the passive carriage by wind currents, or by other organisms (*e.g.* other invertebrates, amphibians, rodents, etc.), two tests were conducted using the setup of experiment 1.

In the first test, 250 second or third instar *An. arabiensis* larvae taken from the laboratory colony were introduced in the two breeding habitats with fresh water and soil in both treatment and control SFS chambers, which had been prepared exactly as described for Experiment 1. No adult mosquitoes were released in either chamber. The daily pupation, mortality and emergence rates were recorded until all pupae were dead or had emerged as adults. The experiment was allowed to run until all had pupated.

In the second test, the chambers used for treatment and control were reversed, *i.e.* the control was run in the chamber previously used for treatment and *vice versa*. A total of 5,000 adult female mosquitoes were released in each chamber and two replicates of the second test were conducted and breeding habitats productivity were monitored as described in experiment 1.

### Experiment 3: Mosquito resting site preference inside the semi field systems

To determine the proportions of released mosquitoes that rested inside the clay pots in the experimental setup, adult female mosquitoes were released inside treated and control SFS chambers, as described for experiment 1. On each morning over the following three days (an average period for eggs development before mosquito visits the habitats to lay eggs), all mosquitoes found resting inside clay pots and walls and ceiling of the cattle hut were collected using mouth aspirators, counted and recorded as either fed or unfed. The experiment was repeated twice, first with 2,000 mosquitoes and then with 4,000 mosquitoes released in each chamber (released mosquitoes were increased in the second replicate to increase the proportion of mosquitoes to be recaptured).

### Experiment 4: Determining contamination rates of the Anopheles arabiensis population resting inside clay pots

To estimate the proportion of *An. arabiensis* contaminated with PPF in this setup, 5,000 unfed adult female mosquitoes were released inside both treated and control SFS chambers, where only clay pots were treated with PPF as described in experiment 1. On each of the three mornings after release, a maximum of 60 mosquitoes (30 from each of the resting sites) were collected inside all clay pots and mud huts (walls and ceiling) and assessed for their feeding status. Following resting behaviour in mosquito after acquiring a blood meal, mosquitoes were collected 36 hrs after release to ensure that high proportion was blood-fed. Individual mosquitoes were collected with separate mouth aspirators and held in a plastic cup (approximately 30 – 60 minutes) to avoid cross-contamination until use. Mosquitos were killed by refrigeration and each mosquito was suspended in 50 ml of water containing 10 third stage larvae of laboratory-reared *An. arabiensis* to monitor larval mortality and pupa emergence inhibition, over 12 days. In addition, the plastic collection aspirators were rinsed with water to remove any possible PPF particles and clean water added to a total volume of 50 ml in which 10 third-stage larvae were suspended, and followed up as just described. The experiment was repeated twice.

To calculate the proportion contaminated, a maximum mortality threshold above an upper 95% CI from a control section was set. Thus an observed larval or pupal mortality in a bioassay cup above the set threshold in the treatment arm, implied that the suspended mosquito was contaminated. The estimated contamination in the treatment section was corrected using Abbot’s formula [30], where the control larval mortality was greater than 5%. Corrected contamination = [% Contamination –% mortality in control)/(100 –% mortality in control)] × 100.

### Experiment 5: Determination of autodissemination efficiency with fewer treatment points and more breeding habitats

The impact of few treated clay pots (1–2) with PPF to deliver PPF contamination to resting mosquitoes was determined in two tests. In the first test, only two of the eight pots were treated with PPF and compared to a control section where all eight pots remained untreated. A batch of 5,000 unfed female *An. arabiensis* were released once in a control and treatment chambers.

In the second test, only one pot was treated with PPF in treatment section, and 5,000 unfed female *An. arabiensis* were released in a control and treatment chambers, in three consecutive batches of 2,000, then 2,000 and lastly 1,000, with an interval of one day between releases. The rationale of releasing different mosquito batches was to facilitate multiple visiting events of mosquitoes to the habitats, which were likely to occur mosquitoes are released in different batches rather than single batch. This also mimic what is likely to happen in nature where different mosquitoes are likely to transit in the same clay pots over time.

In both tests, six breeding habitats were provided, and pupae collected from individual habitat were monitored as described until all were dead or had emerged as adults.

### Data analysis

All data were analysed using R v2.12.2 [31] and the lme4 package [32] for generalized linear mixed effects models. The differences in the total number of pupae collected and proportion emerged between control and treatment SFS chambers were determined with Poisson and binomial distribution respectively using a best-fit generalized linear mixed effect model. While treatment groups (with/without PPF) were classified as fixed effect in the model, experimental replicates, numbers of mosquito released, numbers of larvae, total numbers of pupae collected per control and treatment chambers, and numbers of breeding habitats per control and treatment chambers were assigned as random effects for the autodissemination of PPF and larval bioassay data.

## Results

### Experiment 1: Evaluation of PPF-treated clay pots for the delivery of pyriproxyfen to resting adult female mosquitoes for subsequent autodissemination

**Figure 2:**
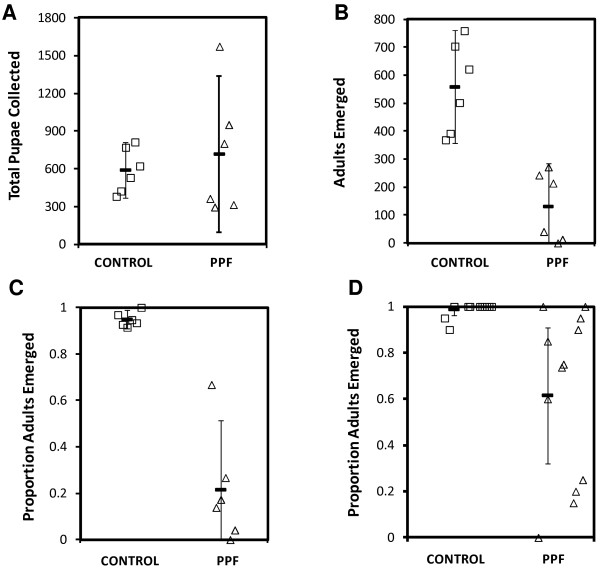
Number of pupae produced (A), adults emerged (B), proportion of adult emerged (C) in the breeding habitats and proportion of adult emerged from larval bioassay on water samples from control and PPF - treated sections (D).

The results of the experiments measuring the impact of PPF-treated resting pots on emergence from nearby breeding habitats are summarized in Figure 2. In the six replicates carried out, an average proportion (95% CI) of adult emerged per experimental replicate was 0.95 ± 0.39) in the control group compared to 0.21 ± 2.99) in the PPF treatments (*p* < 0.0001) (Figure 2C). There was no difference in the mean number (95% CI) of pupae collected from the treatment group (717 ± 622.8) compared with the control group (590 ± 220.9) (*p* = 0.579) (Figure 2A), suggesting that oviposition behaviour of mosquitoes after PPF treatment was not affected by the treatment. However, mean (95% CI) proportion of adult emerged from collected pupae were significantly high in the control group (558 ± 201.9) compared with the treatment group (130.5 ± 155.6) (*p* < 0.0001) (Figure 2B). Low adult emergence rate observed in the treatment chambers strongly suggest the occurrence of PPF autodissemination events mediated by gravid female mosquitoes attempting to oviposit.

In the laboratory bioassay measuring the effect of breeding habitat water on development of larvae, an average proportion (95% CI) of 0.987 ± 0.02 emerged to adults in water from the controls, while only 0.62 ± 0.29 emerged from the treatment group (*p* = 0.0003), (Figure 2D).

In the second larval bioassay, laboratory-reared larvae placed in the breeding habitats after the clay pot experiment ended, had significantly lower average (95%CI) emergence proportion in the treatment chamber (0.16 ± 0.23) compared to the control chamber (0.97 ± 0.05) (*p* < 0.0001), which confirm auto dissemination of PPF to the breeding sites. Attrition of introduced larvae due to predation and other natural causes were similar in both groups (315/500 and 359/500 larvae accounted for in control and treated groups respectively) and there was no evidence of any increase in larval mortality due to PPF *(p* = 0.773). All introduced larvae emerged successfully or died within 20 days of the start of the experiment.

### Experiment 2: Confirmation that pyriproxyfen contamination of breeding habitats was mosquito-borne

In the first test of experiment 2 carried out, laboratory-reared larvae were placed in the breeding habitats of control and treatment chambers, prepared as described for experiment 1, except that here, no mosquitoes were released. The result of the single replicate showed that there was no difference in average (95% CI) proportion adult emergence per day between treatment (0.63 ± 0.24) and control sections (0.69 ± 0.32), (*p* < 0.0001). The total number of pupae collected from breeding habitats in the control (n = 379) and treatment (n = 392) chambers were not different (*p* > 0.05).

In the second test of experiment 2, the design of experiment 1 was repeated by releasing 5,000 adult female mosquitoes in each experimental chamber except here, the control was run in an SFS chamber previously used for PPF treatment, and *vice versa* for the treatment. Average (95% CI) adult mosquito proportion emergence were significantly higher in the control group, both before (0.95 ± 0.39) and after (0.72 ± 0.34) the locations were switched compared to the treatment (0.21 ± 2.99) and (0.05 ± 0.07) (*p* < 0.0001). The results of both experiments demonstrated that reductions in emergence rates in the breeding habitats occurred only when adult mosquitoes were present in the PPF-treated chamber.

### Experiment 3: Mosquito resting site preference inside the semi field systems

**Figure 3:**
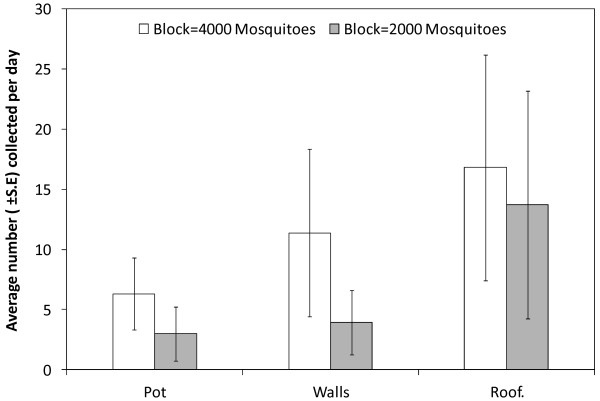
Average number of mosquitoes collected at different resting sites inside the Semi Field Systems.

All recaptured mosquitoes from different resting sites were blood fed. A mean (95% CI) recapture rate of (0.385 ± 0.02) was achieved in all of the replicates carried out, with no difference seen between control (0.38 ± 0.005) and treatment groups (0.39 ± 0.021). (*p* = 0.266). Although, total number of mosquitoes recaptured increased when the number of mosquitoes released was greater (*p* = 0.006), the proportion of mosquito recaptured remains similar between replicates (*p* = 0.543). As Figure 3 shows, the majority of mosquitoes were collected from the ceiling and walls within the hut with 17% found within the resting pots.

### Experiment 4: Determining contamination rates of Anopheles arabiensis population resting inside clay pots

As determined by their ability to inhibit adult emergence in a laboratory bioassay, 100% of all mosquitoes collected inside treated clay pots were found to be PPF-contaminated, while approximately 72% of those found resting in the hut within the treated chamber, were contaminated. Mosquitoes from PPF treated clay pots and huts caused (0.005 ± 0.007) and (0.52 ± 0.06) average adult emergence proportion from exposed larvae respectively in larval bioassay. In the control chamber, an average (95% CI) of (0.925 ± 0.08) of all larvae successful emerged to adults during larval bioassay using mosquitoes collected from clay pots and cattle shed in the control chamber.

### Experiment 5: Determination of autodissemination efficiency with fewer treatment points and more breeding habitats

In both tests, impacts of PPF on pupal emergence were observed in all habitats in the treated chambers. When two clay pots were tested, the mean (95% CI) pupae collected from all breeding habitats were similar between control (52.57 ± 26.98) and treatment (62.92 ± 34.15) chambers, (*p* = 0.522). Similarly, the mean number of pupae collected was not different between control (100.34 ± 19.65) and treatment (104.88 ± 23.66) chambers when one clay pot was tested (*p* = 0.883). The mean proportion (95% CI) of emerged adults was significantly reduced in the treated chambers when two (0.33 ± 0.18) or only one (0.34 ± 0.13) clay pots were treated compared with the respective controls (0.82 ± 0.12); (0.98 ± 0.02); *p <* 0.0001)).

## Discussion

Previous field studies have demonstrated the potential for the autodissemination technique when applied to free flying population *Aedes* mosquito species under field settings [21,22]. In this study, we also proved the occurrence of PPF autodissemination using captive populations of malaria vector *An. arabiensis* under semi-field settings. Overall, autodissemination of PPF by *An. arabiensis* inhibited 82% of adult emergence, which is compatible with the control level of 80% recommended by WHOPES for controlling malaria vector juvenile stages [33] under semi-field conditions. In some cases, for example experiment 1, Figure 2C, total emergence inhibition in PPF-treated sections was achieved with no single adult mosquito emerging from these habitats. Larval bioassays showed a significantly lower adult emergence rate in the treatment sections compared to the control further confirming the delivery of PPF to the breeding habitats in all experiments. More importantly, by introducing insectary larvae directly in the habitats, an even lower emergence rate was observed compared to the control sections. This could be due to the presence of organic matter in the breeding habitats that would allow PPF adsorption and could prolong its persistence in aquatic habitats [34].

Though not clearly elucidated by the data presented here, it remains as a limitation of current study, that wide range and many number of mosquito released (1,500 – 5,000) in relation to number and size of breeding habitats might have affected the productivity of the habitats provided (pupae as a proxy indicator) by causing high larval mortality in the habitats due to overcrowding factors [35], and result in relative small number of pupae collected. However, the reason for a wide range was due to shortage of mosquitoes with a same age whereas many mosquitoes were released to make sure that our experiments were not confounded by shortage of mosquitoes following natural mortality and scavenging. Surprisingly, variations in the numbers of mosquitoes released did not affect the proportions of adults that ultimately emerged from the pupae in the contaminated breeding habitats, the inclusion of the numbers of mosquitoes released resulted in the best model. Since the numbers of mosquitoes visiting contamination stations would have differed between experiments and replicates, variation in mosquito numbers released and pupae collected from the were qualified as random rather than fixed factor.

Importantly, similar emergence rate recorded in the absence of mosquitoes between control and treatment chambers in first test of experiment 2 indicate that passive transfer of PPF (which might have confounded or potentially artificially enhanced any observed impact) did not occur at any stage in these studies. In addition, similar impact of PPF on adult emergence observed in the second test of experiment 2 as the results of released mosquitoes before and after switching locations of control and treatment chambers confirmed that dissemination by ovipositing mosquitoes alone was responsible for transfer of the effective dosages of PPF to the breeding habitats.

In assessing potential mosquito resting sites for targeting with PPF inside SFS, similar number of mosquitoes recaptured between control and treatment groups indicated that PPF does not repel resting mosquitoes. Overall, the proportions of recaptured adult female mosquitoes were few; this might have been caused by restricted collections from few designated places, and missed those resting in the vegetation grown inside the experimental chambers. High resting preference of mosquitoes to the wall and ceiling of the mud hut compared to the clay pots, highlight the potential of targeting these sites with PPF.

The results of experiment 5 are of particular importance because they demonstrated that only one treated resting pot competing with alternative untreated resting sites including seven clay pots and resting sites within the mud hut was sufficient to inhibit > 65% adult emergence in six breeding habitats via ovipositing mosquitoes alone. These findings are very promising and highlight the potential that autodissemination offers for amplification of limited numbers of treatment points to significant levels of effective breeding habitat treatment coverage. Clearly, field-based experiments and mathematical modelling should now be designed to investigate this further and establish the relationship between contamination stations and habitats coverage.

The mechanism of PPF delivery to mosquitoes is crucial for the overall success of the autodissemination technique [21-23]. In this study, the use of clay pots as a point source for PPF application effectively delivered PPF to the mosquitoes resting within and at rates sufficient to enable autodissemination. The attractiveness and usefulness of clay pots as an outdoor and indoor sampling tool for malaria and other disease vectors as well as a delivery tool for entomopathogenic fungi has been described elsewhere [28,36,37]. Although absolute numbers of mosquitoes resting inside clay pots are relatively low, these tools are considered to be efficient for sampling blood fed mosquitoes compared to many other sampling techniques [29]. The results presented here indicate that this simple and affordable method has additional potential in vector control.

When aquatic habitats are limited, the minority of mosquitoes that are contaminated in clay pots and then carry lethal doses of PPF to their aquatic habitats also affect the offspring of uncontaminated mosquitoes. Thus, contaminated adults amplify the impact of their own contamination by affecting the offspring of all mosquitoes that share the contaminated mosquito’s breeding site [38,39]. Although not investigated in this study, field deployment of autodissemination approach is predicted to be affected by number of mosquitoes visiting the habitats, size of the breeding habitats and distance of the habitats from PPF contamination stations. Moreover, targeting only the clay pots with PPF resulted in the effective contamination of mosquitoes that were ultimately collected from the huts, suggesting that blood-fed mosquitoes move between resting sites during that phase of their gonotrophic cycle. This is clearly an advantage in terms of optimizing the effect of PPF through further “coverage amplification of the habitats” whereby PPF is likely to be delivered to many breeding habitats by PPF-contaminated mosquitoes using few habitats, and potentially might act to reduce the number and costs of contamination stations required [40]. Clay pots, by providing shelter from rain and sunlight, might also prolong the lifespan of single PPF treatments, an important consideration in any ‘insecticide’-based program. However, it should be noted that this experimental design provides only estimates, rather than actual numbers, of mosquitoes that rest or pass through clay pots and of whether they are contaminated or not.

The impact of PPF varies at different stages of the mosquito’s life cycle. Previous work has shown that mosquitoes that are contaminated within 24 hrs of a blood meal become sterilized and do not lay eggs [41,42] but this sterilization effect does not occur when exposure to PPF occurs beyond 24 hrs after the blood meal. However, in the experiments reported here, the test mosquitoes produced large numbers of developing offspring in the artificial habitats provided, suggesting that the clay pots set outside the cattle sheds, were not visited by blood-fed mosquitoes until sometime after completion of feeding when egg-maturation was underway. If so, then it was while resting outdoors after the blood meal that these mosquitoes were contaminated, and targeting this stage of the gonotrophic cycle (i.e. >24 hours after blood feeding) may maximize delivery of PPF to the breeding habitats [23]. Alternatively, if PPF-contamination occurred immediately after or within 24 hours of blood feeding, then it suggests that these PPF-sterilized mosquitoes, despite not being gravid, went on to visit the breeding habitats where they prevented emergence of the next generation of mosquitoes from the eggs laid by uncontaminated adults.

Although a key necessity for its success is the development of efficient contamination stations, a role performed very well by the clay pots in the experiments reported here, the autodissemination technique potentially can target both indoor and outdoor biting mosquitoes, susceptible and pyrethroid resistant mosquito strains at their larval habitat, with impacts on adult mosquito density and malaria transmission [14,40,43]. The integration of this method of control with current vector control measures (LLINs and IRS) could help in the control of outdoor biting vectors such as *An. arabiensis* as well as providing an approach to managing insecticide resistance [44]. The autodissemination of insecticides by adult mosquitoes for the control of malaria is likely to work better in the dry season when the breeding habitats are few and stable with reduced water flushing [38,40]. With recent development of highly potent formulation up to 10% AI PPF dust, which is effective at ultra-low dose, it might be possible to effectively contaminate greater volumes than current possible using malaria vectors and other mosquitoes that share the habitats with Anopheles mosquitoes.

This is the first study to investigate the potential for using PPF autodissemination for the control of *An. arabiensis,* one of the efficient African malaria vectors. The results are very promising and indicate that this approach offers an opportunity to be considered amongst future malaria control strategies in Africa. Before its full potential can be assessed, further vector studies will be required in key areas: 1) the effectiveness seen in these semi-field experiments must be demonstrated under full field conditions; 2) quantitative studies on ‘amplification’ are required to determine the numbers and densities of treatment points required to deliver effective control at breeding sites; 3) investigations of impacts on other species sharing the breeding sites, including other vectors, nuisance mosquitoes and non-target species.

## Competing interests

The authors have declared no competing interests.

## Authors’ contributions

DWL, CH, SD, SM, PJM and GJD proposed the study hypothesis and experimental design. DWL, SSK and CH performed statistical analysis and wrote the first draft of the manuscript. DWL supervised the experiments and data collection. DWL, SM and PJM wrote the manuscript. All authors read and approved the final manuscript.
